# Microtubule acetylation is not required for HIV-1 infection or TRIM69-mediated restriction of HIV-1 infection

**DOI:** 10.1128/jvi.01026-24

**Published:** 2025-01-13

**Authors:** Drew M. Lichon, Natalie J. LoMascolo, Bryan C. Mounce, Edward M. Campbell

**Affiliations:** 1 Department of Microbiology and Immunology, Loyola University Chicago 548051 , Maywood, Illinois, USA; The Ohio State University, Columbus, Ohio, USA

**Keywords:** HIV-1, microtubules, acetylated microtubules, TRIM69

## Abstract

Microtubule acetylation, a post-translational modification catalyzing the addition of acetyl groups to lysine residues on alpha tubulin, confers mechanical resilience to microtubules and influences intracellular cargo transport. Despite its known cellular functions, its role in viral infections remains poorly understood. The goal of this study was to determine the role of microtubule acetylation in both HIV-1 infection and TRIM69-mediated restriction. To this end, we generated CRIPSR/Cas9 vectors to disrupt alpha-tubulin acetyltransferase (αTAT1), the main enzyme responsible for microtubule acetylation. We assessed the role of acetylation in HIV-1 infectivity and the degree to which TRIM69 relies on microtubule acetylation for its ability to restrict HIV-1. We determined that microtubule acetylation is not required for HIV-1 infection and that preventing microtubule acetylation actually leads to a modest increase in HIV-1 infection. We also determined that TRIM69 can restrict a diverse range of viruses and that its restriction of HIV-1 does not rely on microtubule acetylation.

**IMPORTANCE:**

Although microtubule acetylation is a well-studied post-translational modification in the context of cellular processes, its role during viral infections remains underexplored. Existing studies often rely on various protein and drug perturbations to indirectly examine microtubule acetylation. In this study, we directly target the enzyme responsible for microtubule acetylation to delineate its role in both HIV-1 infection and TRIM69-mediated restriction.

## INTRODUCTION

The human immunodeficiency virus type 1 (HIV-1) continues to pose a significant global health threat, with over 38 million individuals living with HIV worldwide. Understanding the mechanisms by which HIV-1 interacts with the host cell during replication is paramount in devising effective therapeutic strategies. The ability of HIV-1 to interact with proteins of the microtubule network is known to be critical during infection (1–3).

Microtubules, composed of α- and β-tubulin heterodimers, serve as critical conduits for intracellular transport and play a pivotal role in orchestrating various cellular processes (4). Post-translational modifications, such as acetylation, regulate microtubule stability and dynamics, thereby influencing their cellular functions (5, 6). The process of acetylation involves the addition of an acetyl group to lysine residues located on the α-tubulin subunit of microtubules. This modification is catalyzed almost singularly by the enzyme alpha-tubulin acetyltransferase (αTAT1) (7–10). Acetylation occurs on the luminal surface of the microtubule, stabilizing its structure in two separate ways (11–13).

The stability of acetylated microtubules manifests in two central forms: increased mechanical strength, which is specific to microtubule acetylation, and enhanced resistance to depolymerization, which requires the accumulation of both internal and external post-translational modifications (PTMs) on microtubules. For instance, herpesviruses, such as HSV-1, utilize surface PTMs on long-lived stable microtubules to affect kinesin-1 affinity and enhance cargo transport (14). This interaction facilitates the efficient intracellular trafficking of the virus. In contrast, tubulin acetylation also confers mechanical strength and stability, which is crucial for the intracellular movement of large organelles, such as the nucleus, by cytomegalovirus (15, 16). This process is related to the force exertion capability provided by tubulin acetylation rather than its role in cargo transport.

Recent studies have highlighted the critical role of acetylated microtubules in the life cycle of several viruses, particularly influenza A virus (IAV) and Epstein–Barr virus (EBV). In the case of EBV, microtubule acetylation facilitates viral evasion of innate immune responses, enhancing the virus’s ability to persist within the host (17). Similarly, IAV leverages microtubule acetylation to promote increased virion release from infected cells, thereby boosting viral replication and dissemination (18). Sabo et al. were the first to observe microtubule acetylation following HIV-1 infection, observing that as early as 2 h post-infection there is a robust upregulation of this PTM (19). Furthermore, this study demonstrates how specialized microtubule-binding proteins facilitate the stabilization of microtubules during HIV-1 infection. Consistent with this, we have also previously observed microtubule acetylation during HIV-1 infection and observed that preventing microtubule acetylation through the use of an mTOR inhibitor, which depletes cellular acetyl-CoA pools, inhibits HIV-1 infection (20).

TRIM69 has emerged as a pivotal player in the host innate immune response and antiviral defense mechanisms. TRIM69, belonging to the C-IV subfamily, which also includes the well-studied TRIM5α (21). Structurally akin to other TRIM proteins, TRIM69 comprises a RING, B-box, and coiled-coiled domain followed by a PRY-SPRY domain. Previous publications have highlighted the multifaceted role of TRIM69 in regulating viral infection, including its impact on viral replication, innate immune sensing, and host defense mechanisms in multiple viruses, including vesicular stomatitis virus (VSV), dengue virus, and SARS-CoV-2 (22–24). Recent investigations have uncovered a relationship between TRIM69 and acetylated microtubules, with a recent study providing evidence that TRIM69 induces microtubule acetylation in the context of inhibiting HIV-1 infection (25).

To more definitively understand the role of microtubule acetylation on HIV-1 infection and in TRIM69-mediated HIV-1 restriction, we examined both HIV-1 infection and TRIM69-mediated restriction of HIV-1 in cells lacking αTAT1, the acetyltransferase responsible for microtubule acetylation (5, 7, 8, 10). As expected, we observed that αTAT1 knockout prevented microtubule acetylation induced by HIV-1 infection. However, we observed that αTAT1 knockout did not negatively influence HIV-1 infection, and instead led to an increase in HIV-1 infection. We also examined the ability of TRIM69 to inhibit HIV-1 infection in the context of αTAT1 knockout. Similarly, our results demonstrated that TRIM69 can still restrict HIV-1 in the absence of microtubule acetylation. Notably, although TRIM69 associates with acetylated microtubules, microtubule acetylation is not required for its restriction activity, suggesting an independent role for TRIM69 in both inducing microtubule acetylation and restricting viral infections. Finally, we observed that TRIM69-mediated restriction extends beyond HIV-1 to encompass other viruses, such as Rift Valley fever virus strain MP12 (MP12) and Coxsackie virus B3 (CVB3).

## RESULTS

### Microtubule acetylation is not required for HIV-1 infection

**Fig 1:**
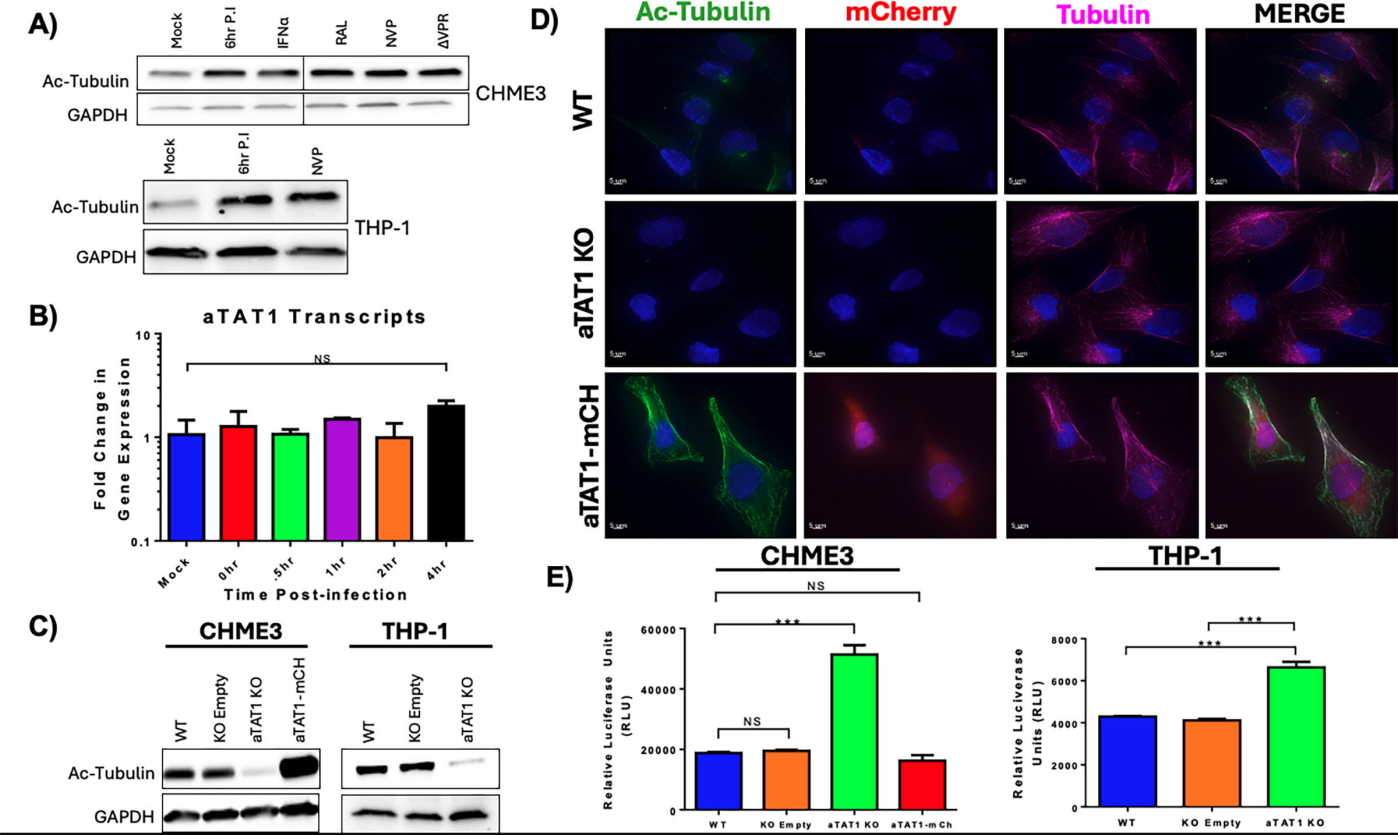
Microtubule acetylation is not required for HIV-1 infection. (A) CHME3 (Top) and THP-1 (Bottom) cells were synchronously infected with VSV-g pseudotyped HIV-1, with or without the presence of HIV-1 VPR protein, followed by treatment with interferon-α, raltegravir, or nevirapine. Cell samples were collected at specified time points and analyzed for levels of acetylated tubulin and GAPDH. (B) THP-1 cells were infected with VSV-g pseudotyped HIV-1 and collected at given time points. RNA was isolated, converted to cDNA, and assayed for αTAT1 transcript levels using αTAT1 specific primers. Error bars represent ± standard error of the mean (SEM). Statistical significance was determined by a one-tailed *t*-test, with *P* < 0.05 considered significant. (C) CHME3 (Left) and THP-1 (Right) cells were stably transduced with either an empty V2 CRISPR/Cas9 construct, a V2 CRISPR/Cas9 construct targeting αTAT1, or a pLVX construct containing the open reading frame for aTAT1 with an N-terminal mCherry fluorophore tag. Cell lysates were collected and assayed for protein levels of acetylated tubulin and GAPDH. (D) Cells were transduced with the indicated aTAT1 construct and subjected to immunofluorescence staining for acetylated tubulin, alpha tubulin, and nuclear staining (DAPI). Error bars represent ± standard error of the mean (SEM). Statistical significance was determined by a one-tailed *t*-test, with *P* < 0.05 considered significant. (E) CHME3 (Top) and PMA-differentiated THP-1 (Bottom) cells were infected with an HIV-1-GFP reporter virus. At 48 h post-infection, infectivity was determined by flow cytometry analysis to quantify the percentage of GFP-positive cells.

To establish cellular models to understand the role of microtubule acetylation on HIV-1 infection, we monitored microtubule acetylation induced during HIV-1 infection of CHME3, a microglial cell line, and THP-1 cells, a monocytic cell line, which can be terminally differentiated into a macrophage-like phenotype using phorbol 12-myristate-13-acetate (PMA). Following infection, cells were collected, and microtubule acetylation was assessed by Western blot. Consistent with previous findings (19), HIV-1 infection induced microtubule acetylation 6 h post-infection (Fig. 1A). Neither the reverse transcriptase inhibitor nevirapine nor the integrase inhibitor raltegravir affected the formation of stable microtubules, revealing that microtubule acetylation is not dependent on reverse transcription or integration (Fig. 1A). Moreover, infection with a pseudotyped HIV-1 virus lacking the Vpr protein (∆Vpr) still induced the formation of acetylated microtubules (Fig. 1A), indicating that VPR does not impact microtubule acetylation. The observed increase in microtubule acetylation occurred in the absence of transcriptional upregulation of αTAT1 (Fig. 1B).

To understand the consequences of microtubule acetylation during HIV-1, we generated CRISPR/Cas9 vectors to disrupt the αTAT1 gene and generated an expression system expressing αTAT1 fused to mCherry (referred to as αTAT1 KO and αTAT1-mCh) and transduced THP-1 and CHME3 cells with these vectors (Fig. 1C). Although αTAT1 knockout could not be assessed by monitoring αTAT1 expression due to a lack of reliable antibody, cells transduced with the αTAT1-targeted CRISPR gRNA exhibited a marked reduction in microtubule acetylation, whereas we observed overexpression of αTAT1-mCh, leading to a marked hyperacetylation of microtubules (Fig. 1C and D).

We therefore used these cells to determine the role of microtubule acetylation on HIV-1 infection. Upon HIV-1 infection, cells lacking αTAT1 exhibited approximately a twofold increase in HIV-1 infection compared with control cells (Fig. 1E). Conversely, overexpression of αTAT1 did not alter HIV-1 infectivity. These findings demonstrate that microtubule acetylation is not required for HIV-1 infection.

### aTAT1 knockout reduces the formation of stable microtubules following HIV-1 infection

**Fig 2:**
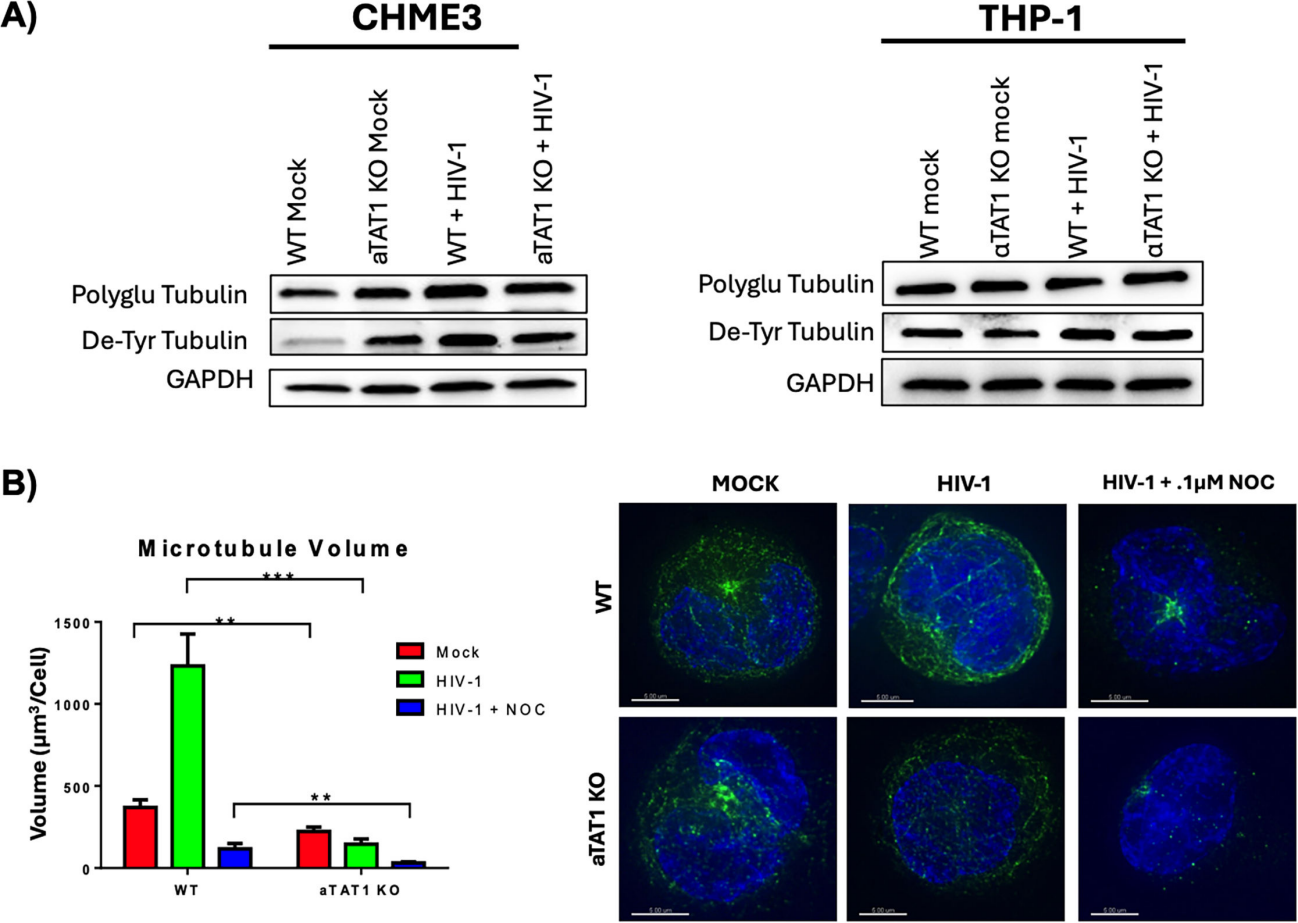
αTAT1 knockout does not increase the formation of stable microtubules following HIV-1 Infection. (A) CHME3 (left) and THP-1 (right) cells were stably transduced with a CRISPR/Cas9 construct targeting αTAT1. Following αTAT1 knockout, cells were mock-infected or infected with HIV-1. Six hours post-infection, cell lysates were collected and analyzed for protein levels of polyglutamylated tubulin and detyrosinated tubulin compared with wild-type controls. (B) Wild-type and αTAT1 knockout THP-1 cells were infected with HIV-1. Five hours post-infection, cells were either mock-treated or treated with nocodazole (0.1 µM) for 1 h. Fixed coverslips were stained for alpha tubulin and imaged, and microtubule volume was quantified using IMARIS software across 10 + images. Quantified microtubule volume is shown on the left, and representative images are shown on the right.

To assess the effects of αTAT1 knockout on microtubules, we investigated the post-translational landscape of microtubules in αTAT1 knockout (KO) cells. In CHME3 microglial cells, αTAT1 knockout resulted in an increase in detyrosinated tubulin when compared with wild-type cells in the absence of HIV-1 infection (WT) (Fig. 2A, left). After HIV-1 infection, WT CHME3 cells displayed increased levels of both polyglutamylated and detyrosinated tubulin. In contrast, HIV-1 infection did not induce further changes in these tubulin modifications compared with the uninfected aTAT1 KO cell line (Fig. 2A, left). A different result was observed in THP-1 cells, as neither αTAT1 KO nor HIV-1 infection significantly altered these post-translational modifications individually or in combination (Fig. 2A, right).

To further examine microtubule stability in the absence of acetylation during HIV-1 infection, we treated both wild-type and αTAT1 KO THP-1 cells with 0.1 µM nocodazole 5 h post-infection. Immunofluorescence microscopy showed that αTAT1 KO THP-1 cells had a reduced volume of stable microtubules compared with wild-type cells (Fig. 2B and C). These findings suggest that the absence of acetylation leads to a decrease in microtubule stability during HIV-1 infection.

### TRIM69 restriction occurs independently of microtubule acetylation

It has recently been reported that TRIM69 expression leads to an increase in microtubule acetylation, suggesting that the mechanism by which TRIM69 can inhibit HIV-1 infection may be related to microtubule acetylation (25).

**Fig 3:**
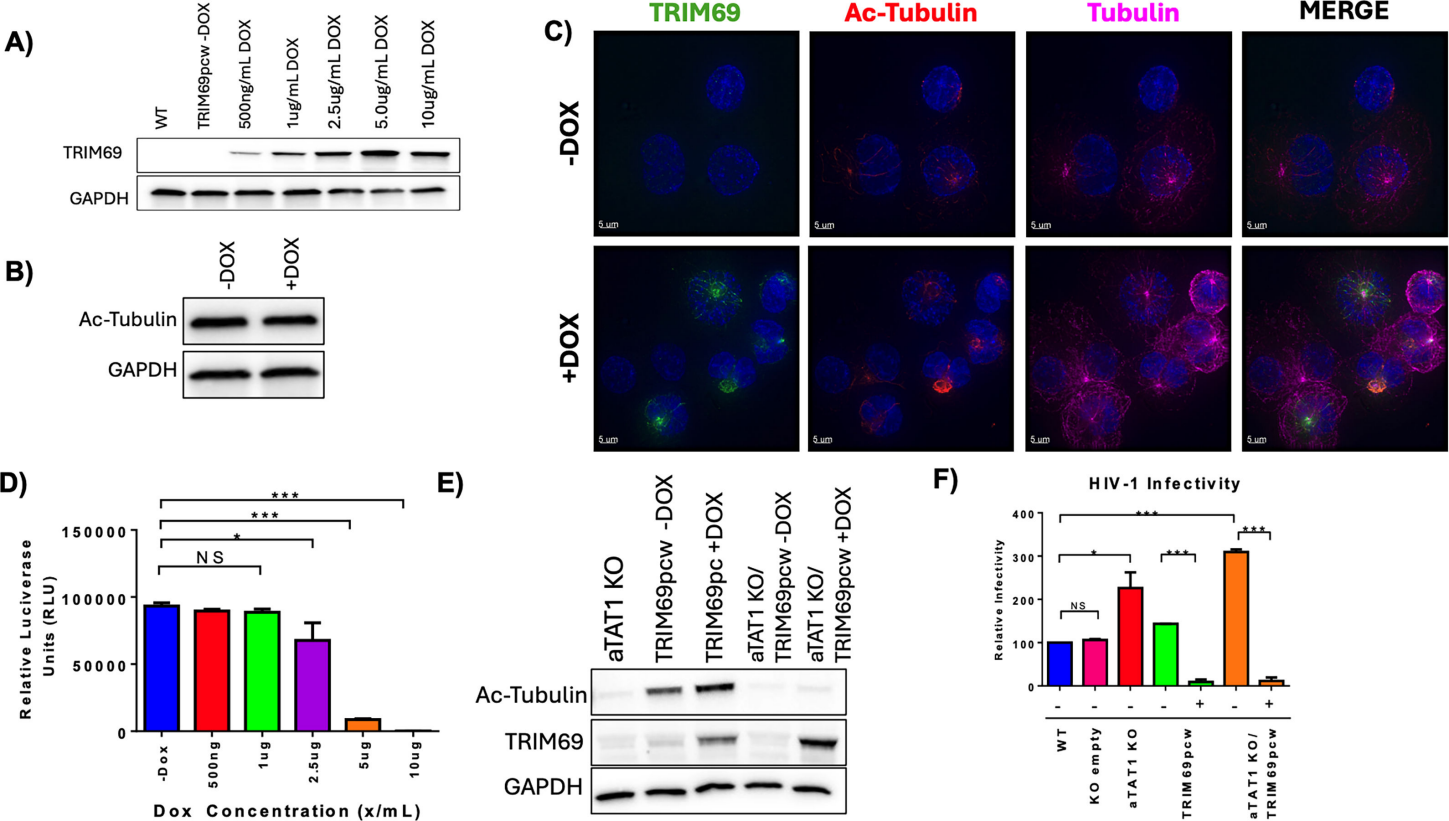
TRIM69 restriction occurs independently from microtubule acetylation. (A) THP-1 cells were stably transduced with a doxycycline-inducible TRIM69 construct and treated with varying concentrations of doxycycline (DOX) for 48 h. Both wild-type (WT) and doxycycline-treated cells were collected and analyzed for protein levels of TRIM69 and GAPDH. (B) Wild-type THP-1 cells were either mock-treated or doxycycline-treated at 5 µg/mL for 48 h. Following treatment, cells were collected and analyzed for protein levels of acetylated tubulin (Ac-Tubulin). As a loading control, protein levels of GAPDH were examined. (C) THP-1 cells were stably transduced with the TRIM69 pcw57 construct and either mock-treated or treated with doxycycline at 5 µg/mL for 48 h. Following treatment, cells were stained and imaged for TRIM69, acetylated tubulin, alpha tubulin, and nuclear staining (DAPI). (D) Transduced THP-1 cells were differentiated with PMA and treated with varying concentrations of doxycycline for 48 h. Subsequently, cells were infected with equal amounts of a firefly luciferase-containing HIV-1 reporter virus. At 48 h post-infection, cells were analyzed *via* luciferase assay to determine infectivity levels. (E) THP-1 cells were stably transduced with either the aTAT1 V2 CRISPR/Cas9, TRIM69 pcw57, or both constructs. After selection, cells were treated with 5 µg/mL of doxycycline for 48 h. Subsequently, cells were lysed and assayed for protein levels of acetylated tubulin, TRIM69, and GAPDH. (F) THP-1 cells stably expressing an empty V2 CRISPR/Cas9, αTAT1 V2 CRISPR/Cas9, TRIM69 pcw57, or both were differentiated with PMA and treated with 5 µg/mL of doxycycline for 48 h. Following treatment, cells were infected with equal amounts of VSV-g pseudotyped NL4.3 Luc-mCherry HIV-1. At 48 h post-infection, infectivity was determined by flow cytometry analysis to quantify the percentage of mCherry-positive cells. A representative flow experiment is presented on the left, while normalized infectivity across three experiments is provided on the right. Error bars represent ± standard error of the mean (SEM). Statistical significance was determined by a one-tailed *t*-test, with *P* < 0.05 considered significant.

To determine if microtubule acetylation is required for TRIM69-mediated inhibition of HIV-1 infection, we established a doxycycline-inducible TRIM69 overexpression system within THP-1 cells (Fig. 3A). Consistent with the study by Song et al., we observed an increase in microtubule acetylation in cells expressing TRIM69 and observed colocalization of TRIM69 with acetylated tubulin (Fig. 3B and D) (25). When challenged with HIV-1, we observed that increased TRIM69 expression led to a potent inhibition of HIV-1 infection (Fig. 3C).

To explore the potential link between TRIM69 restriction and microtubule acetylation induction, we induced TRIM69 expression in αTAT1 KO THP-1 cells (Fig. 3D). These cells exhibited TRIM69 expression under doxycycline treatment while maintaining the absence of microtubule acetylation associated with the CRISPR/Cas9 knockout of αTAT1. In these cells, HIV-1 infection was still potently inhibited following induction of TRIM69 expression (Fig. 3E), demonstrating that TRIM69-mediated restriction of HIV-1 operates independently of its capacity to induce acetylated microtubule formation.

### TRIM69 restricts HIV-1 at the step of reverse transcription

**Fig 4:**
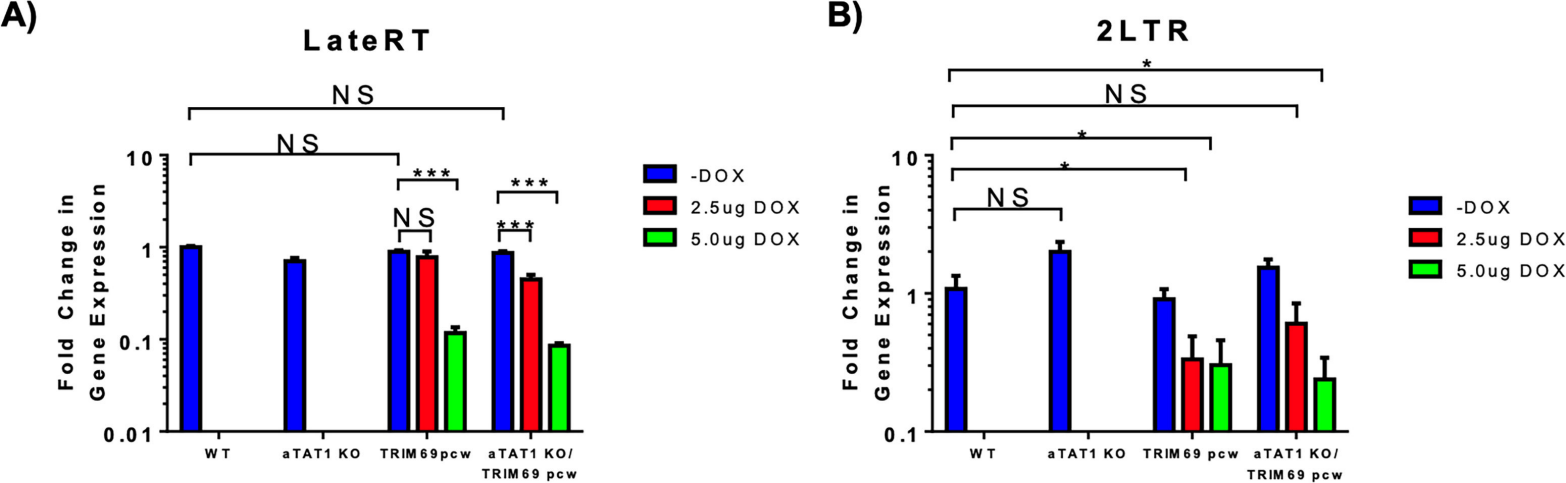
TRIM69 restricts HIV-1 during reverse transcription. THP-1 cells expressing the inducible TRIM69pcw construct were treated with doxycycline for 48 h prior to infection. Following treatment, cells were infected with DNase-treated HIV-1 for 24 h. Following infection, DNA was isolated and subjected to qRT-PCR analysis using primers specific for LateRT products (**A**) or 2LTR aggregates (**B**). Results are normalized to infected wild-type THP-1. Error bars represent ± standard error of the mean (SEM). Statistical significance was determined by a one-tailed *t*-test, with *P* < 0.05 considered significant.

To determine which step TRIM69 restricts HIV-1, we infected THP1 and isolated DNA 24 h post-infection. Consistent with Song et al., we observed that TRIM69 expression resulted in a reduction in the levels of late reverse transcription (Late RT) and 2-LTR circles (Fig. 4A and B). This demonstrates that TRIM69 inhibits HIV-1 infection prior to the completion of reverse transcription and nuclear import.

### TRIM69 exhibits broad antiviral activity

**Fig 5:**
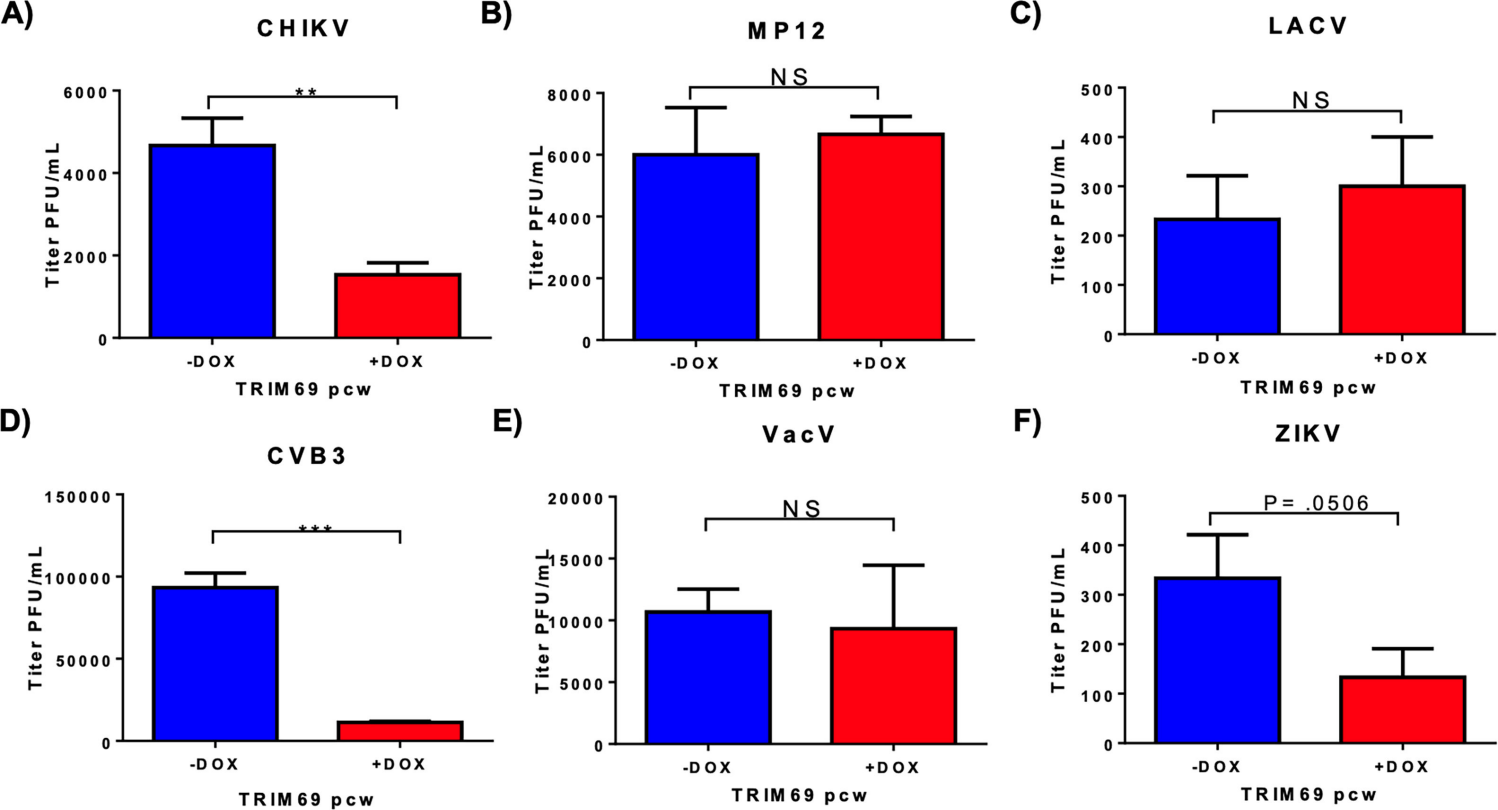
TRIM69 exhibits broad antiviral activity. THP-1 cells expressing the inducible TRIM69pcw construct were treated with 5 µg/mL doxycycline for 48 h prior to infection. Following treatment, cells were infected with an MOI of 1.0 (ZIKV, VACV, and MP12), 0.1 (CHIKV), or 0.01 (LACV and CVB3). Supernatant was collected 48 h post-infection, and viral titer was determined by plaque assay. Error bars represent ± standard error of the mean (SEM). Statistical significance was determined by a one-tailed *t*-test, with *P* < 0.05 considered significant.

To further define the spectrum of viruses susceptible to the antiviral effects of TRIM69, we infected THP-1 cells with chikungunya virus (CHIKV), Rift Valley fever virus (RVFV, strain MP12), Coxsackie virus B3 (CVB3), La Crosse virus (LACV), vaccinia virus (VACV), and Zika virus (ZIKV) in the presence or absence of induced TRIM69 expression. At 48 h following infection, replication was measured via plaque assay. Replication of positive-stranded RNA viruses CVB3, CHIKV, and ZIKV was significantly reduced by TRIM69 expression, while the replication of negative-stranded RNA viruses RVFV and LACV, as well as the DNA virus VACV was unaffected (Fig. 5). These findings demonstrate that the antiviral activity of TRIM69 is broad and capable of inhibiting replication of diverse viruses.

**Fig 6:**
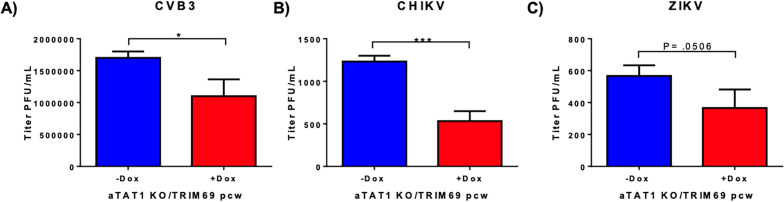
TRIM69 exhibits broad antiviral activity in the absence of microtubule acetylation. THP-1 cells stably expressing a V2 CRISPR/Cas9 construct targeting αTAT1 and cotransduced with an inducible TRIM69pcw construct were treated with 5 µg/mL doxycycline for 48 h prior to infection. Following treatment, cells were infected with an MOI of 1.0 (ZIKV), 0.1 (CHIKV), and 0.01 (CVB3). Supernatant was collected 48 h post-infection, and viral titer was determined by plaque assay. Error bars represent ± standard error of the mean (SEM). Statistical significance was determined by a one-tailed *t*-test, with *P* < 0.05 considered significant.

To assess whether the knockout of αTAT1 affects the ability of TRIM69 to restrict Coxsackie virus B3 (CVB3), chikungunya virus (CHIKV), and Zika virus (ZIKV), we conducted experiments using THP-1 cells with CRISPR-Cas9-mediated knockout of αTAT1. These cells were transduced with a doxycycline-inducible TRIM69 construct and treated with doxycycline to induce TRIM69 expression. Following induction, the cells were infected with CVB3, CHIKV, and ZIKV, and viral replication was assessed using plaque assays. Despite the loss of microtubule acetylation due to αTAT1 knockout, TRIM69 continued to restrict the replication of all three viruses (Fig. 6). These results further demonstrate that TRIM69 employs acetylation-independent mechanisms to exert its broad antiviral activity.

## DISCUSSION

The findings presented in this study shed light on the role of microtubule acetylation on HIV-1 infection and TRIM69-mediated inhibition of viral infection. Contrary to results from other studies, including our own (19, 20), we observe that microtubule acetylation is not required for optimal HIV-1 infection. Rather, we observe that inhibiting microtubule acetylation by CRISPR-mediated knockout of αTAT1 led to a modest but significant increase in HIV-1 infection (Fig. 1E). Notably, previous studies examining the role of microtubule acetylation in HIV-1 infection have shown reduced infection under conditions involving viral mutations and mTOR inhibition, though these interventions may have broader effects on viral infectivity and cell viability. While these findings suggested that microtubule acetylation is important for optimal HIV-1 infection, our experiments using direct αTAT1 knockout to specifically target microtubule acetylation indicate that the observed differences in infectivity may be due to factors beyond acetylation alone, highlighting the complexity of the pathways involved in viral replication. Furthermore, we observed that αTAT1 knockout resulted in reduced levels of stable microtubules following HIV-1 infection, consistent with the role of microtubule acetylation as a key post-translational modification needed to increase microtubule resistance to depolymerization.

Similarly, a previous study characterizing the antiviral activity of TRIM69 observed inhibition of HIV-1 and microtubule acetylation induced by HIV-1 infection (25), suggesting that the mechanism of TRIM69 restriction may be related to its ability to induce microtubule acetylation. We similarly observe that TRIM69 expression induces and localizes to acetylated microtubules (Fig. 3B). However, we found that TRIM69 was able to potently restrict HIV-1 infection even when microtubule acetylation was prevented by αTAT1 KO (Fig. 3). Although we did not further define the mechanism of TRIM69 restriction of HIV-1, its ability to influence replication by diverse viruses, including Coxsackievirus B3, Rift Valley fever virus (RVFV), and Zika virus even in the absence of microtubule acetylation (Fig. 5 and 6), suggests that TRIM69 may not directly bind to viral determinants and may rather perturb cellular processes commonly exploited by viruses during infection. Consistent with this, a recent study has reported that TRIM69 can bind to dynein and thereby modulate centromere dynamics and chromosomal segregation (26). It is therefore possible that TRIM69 inhibits viral infection by interfering with the ability of viruses to exploit microtubule trafficking during infection, although future studies are required to test this hypothesis.

As our data demonstrate that microtubule acetylation is not directly exploited by HIV-1 during infection nor required for TRIM69-mediated restriction of HIV-1, the mechanism by which microtubule acetylation is induced during infection remains unclear. Given that microtubule acetylation is observed in the absence of reverse transcription or integration, it is unlikely that nucleic acid sensing by the host cell is driving this response. Rather, the modest increase in infection observed when this response was inhibited by αTAT1 KO suggests that this response may be antiviral in nature, despite it being unnecessary for TRIM69 restriction.

A key limitation of this study is the narrow focus on microtubule acetylation as the primary post-translational modification examined in the context of HIV-1 infection and TRIM69-mediated restriction. While acetylation is an important modification, other PTMs, such as detyrosination, polyglutamylation, and phosphorylation, also play significant roles in regulating microtubule stability, function, and interactions with motor proteins. While we observed no direct change in both polyglutamylation or detyrosination of α-tubulin following αTAT1 knockout and HIV-1 infection, our study did not specifically perturb these pathways to assess their role in HIV-1 infection or TRIM69 restriction. Additionally, the efficiency of the infection could ultimately impact the ability to detect changes in the PTMs within microtubules. It therefore remains possible that these PTMs, either alone or in combination with acetylation, contribute to viral replication and restriction mechanisms in ways that were not captured in this study. Future research should aim to investigate how these additional modifications might influence both viral infectivity and the antiviral activity of host factors such as TRIM69.

## MATERIALS AND METHODS

### Cell lines

THP-1 cells were acquired from ATCC, while CHME3 microglia were obtained from a collaborator. 293Ts and CHME3 cells utilized in this study were cultured in Dulbecco’s Modified Eagle Medium (DMEM), whereas THP-1 cells were cultured in RPMI medium (Cellgrow). Both media were supplemented with 10% characterized fetal bovine serum (FBS), 1,000 U/mL penicillin, and 1,000 U/mL streptomycin. THP-1 cells were differentiated by treatment with 100 ng/mL phorbol myristate acetate (PMA, Sigma) for 24 h, followed by an additional 24 h incubation in normal media without PMA before conducting experiments.

### Constructs

The TRIM69 pcw57 construct was generated by cloning the human ORF for TRIM69 into the lentiviral backbone PCW57 using restriction enzyme digestion and ligation techniques. For the creation of the αTAT1 V2 CRISPR/Cas9 construct, LentiCRISPRv2 (Addgene plasmid no. 52961), generously provided by Feng Zhang from the Massachusetts Institute of Technology, Cambridge, MA, was employed. The construct was designed with the following guide RNA (gRNA) sequence targeting αTAT1: Forward 5′- CAC CGC ATG AGT CTG TGC AAC GCC A-3′, Reverse 5′- AAA CTG GCG TTG CAC AGA CTC ATG C-3′. The αTAT1–mCherry construct was developed by cloning the human αTAT1 ORF into the lentiviral backbone pLVX, with the mCherry fluorophore fused to the N-terminus of the protein.

### Viral and vector production

To produce HIV-1 particles pseudotyped with VSVg, 293T cells were seeded in a 10 cm dish at 70% confluency and transfected with 2 µg pCMV-VSVg and 8 µg of either R7ΔEnvGFP or NL4.3 Luc-mCherry plasmids using polyethylenimine (PEI, MW 25000 Polysciences). After 24 h, the media were replaced with fresh Dulbecco’s Modified Eagle Medium (DMEM), and at 48 h post-transfection, the media containing viral particles were collected. To generate viral vectors carrying the TRIM69 pcw57, αTAT1 V2, or αTAT1–mCherry construct, 293T cells seeded in a 10 cm dish were transfected with 5 µg of TRIM69 pcw57/αTAT1 V2/or αTAT1–mCherry, 3 µg of the packaging plasmid psPAX, and 2 µg of pCMV-VSVg. Viral supernatants were harvested at 48- and 72 h post-transfection and filtered through a 0.45 µm filter (Millipore). Synchronized infection of cells with viruses and viral vectors was achieved through spinoculation at 13°C for 2 h at 1,200×*g*.

### Infection and enumeration of viral titers

For HIV-1 infection, cells were infected with either the R7ΔEnvGFP or NL4.3 Luc-mCherry reporter virus (as listed in figure legend). At 48 h post-infection, infectivity levels were assessed using either a luciferase assay or flow cytometry to determine the percentage of mCherry-positive cells for the NL4.3 Luc-mCherry virus or flow cytometry to determine the percentage of GFP-positive cells for the R7ΔEnvGFP virus. For the TRIM69/aTAT1 KO infectivity experiments, infection levels were normalized within the linear range of infection to ensure accurate comparison.

ZIKV (strain PLCAL; BEI Resources), VACV (strain WR; provided by Tom Gallagher), and CHIKV (strain 181/25; BEI Resources) were derived from VeroE6 cells. LACV (product no. NR-540; BEI Resources) and RVFV (strain MP-12; provided by Shinki Makino) were derived from Huh7 cells. CVB3 (Nancy strain) was derived from HeLa cells. For infections, unless otherwise noted, the virus was diluted in serum-free DMEM (Sigma-Aldrich) for the following multiplicity of infection (MOI): ZIKV (1), VACV (1), CHIKV (0.1), LACV (0.01), RVFV (1), and CVB3 (0.01). Viral inoculum was overlaid on cells for 30 min, then washed with PBS before medium replenishment. The supernatant was collected 48 h post-infection. For plaque assays, dilutions of cell supernatant were prepared in serum-free DMEM and used to inoculate a confluent monolayer of VeroE6 cells for 10–15 min at 37°C. Cells were overlaid with 0.1% agarose in DMEM containing 2% NBCS. CVB3 and VACV samples were incubated for 2 days, CHIKV and LACV samples were incubated for 3 days, and RVFV and ZIKV samples were incubated for 5 days at 37°C. Following appropriate incubation, cells were fixed with 4% formalin (10% formalin; VWR) and stained with crystal violet solution (10% crystal violet; Sigma-Aldrich). Plaques were enumerated and used to back-calculate the number of plaque-forming units (PFU) per milliliter.

### Antibodies and reagents

The anti-acetylated tubulin antibody (Sigma-Aldrich T7451) was utilized for the assay of acetylated tubulin. Rat monoclonal tubulin antibody, employed for both Western blot and immunofluorescence assays, was procured from Abcam (Ab6161). Mouse monoclonal polyglutamylated tubulin antibody used for Western blot analysis was acquired from Sigma-Aldrich (T9822). Rabbit polyclonal detyrosinated tubulin antibody used for Western blot analysis was acquired from Sigma-Aldrich (AB3201). For TRIM69 detection in both immunofluorescence and western blotting, a rabbit polyclonal antibody (Proteintech 12951–1-AP) was utilized. Additionally, a mouse monoclonal antibody against GAPDH (Santa Cruz Biotechnology, INC Sc 0411) was employed for Western blotting. Secondary antibodies conjugated to a fluorophore, utilized for immunofluorescence studies, were obtained from Jackson Immunoresearch Laboratories. Doxycycline hyclate, used at a concentration of 5 µg/mL unless otherwise specified, was purchased from Sigma-Aldrich. Nevirapine (NVP) and DAPI stain were also acquired from Sigma-Aldrich, while aphidicolin was obtained from Cayman Chemicals.

### Generation of stable cell lines

To establish CHME3 and THP-1 cell lines stably expressing TRIM69, viral vectors generated from the TRIM69 pcw57 construct were employed for transduction. Following transduction, cells were treated with 600 µg/mL geneticin (Gibco) 48 h later to select for the stable transduced population. Additionally, viral vectors produced from the plvx αTAT1–mCherry construct were utilized to transduce CHME3 and THP1 cells. For these cells, selection was performed using 2 µg/mL puromycin (Gibco). Moreover, viral vectors derived from the αTAT1 V2 CRISPR/Cas9 construct were employed to transduce CHME3 and THP1 cells, with selection carried out using 10 µg/mL blasticidin (Gibco).

### Western blotting

Cell lysates were prepared by treating cells with 1× Passive Lysis Buffer for 30 min on ice. After incubation, lysates were centrifuged at 13,000 rpm for 10 min, and the supernatant was collected for Western blot analysis. Briefly, 2× SDS was added to the lysed sample and incubated at 100°C for 5 min. Protein concentration was determined using the Pierce BCA protein assay kit (Thermo Scientific), and equal amounts of protein were loaded onto a 4%–15% gradient gel (Bio-Rad). After electrophoresis, the proteins were transferred to nitrocellulose membranes (Bio-Rad). The membranes were then incubated with specific primary antibodies overnight, followed by incubation with secondary antibodies conjugated to horseradish peroxidase (HRP) (Thermo Scientific). Antibody complexes were visualized using SuperSignal West Femto Chemiluminescent Substrate (Thermo Scientific), and chemiluminescence signals were detected using the FluroChem Imaging system (ProteinSimple).

### Immunofluorescence microscopy

Coverslips were fixed and permeabilized using a solution containing microtubule-stabilizing buffer, PFA, glutaraldehyde, and Triton X-100. Prior to antibody labeling, coverslips were washed in MgPBS containing NaBH4 to reduce any residual-free aldehyde groups. Subsequently, coverslips were incubated with a primary labeling mix consisting of Rat monoclonal antibody for tubulin (Abcam ab6161) and Mouse monoclonal antibody for acetylated-α-tubulin (Sigma-Aldrich T7451) for 1 h at room temperature. After primary antibody incubation, coverslips were washed with PBS, followed by the addition of a secondary antibody labeling mix, which was allowed to incubate for 40 min at room temperature. Finally, coverslips were washed with PBS, mounted, and stored at 4°C. Z-stack images were acquired using a DeltaVision wide-field fluorescent microscope (Applied Precision, GE) equipped with a digital camera (CoolSNAP HQ; Photometrics) and a 1.4 numerical aperture 100× objective lens. Excitation light was generated using a solid-state illumination module (Applied Precision, GE), and images were deconvolved using SoftWoRx deconvolution software. Uniform acquisition parameters were applied to all images. Data sets were analyzed and assembled using Prism, version 6.

For the analysis of stable microtubule formation, cells were spinoculated at 13°C for 2 h at 1,200×*g* HIV-1 to ensure synchronized infection. After spinoculation, cells were incubated in normal media for 5 h at 37°C, followed by a 1 h incubation with media containing nocodazole at a concentration of 0.1 µM. Post-incubation, cells were fixed, permeabilized, and stained for tubulin as described in the protocol above. Tubulin volume was analyzed using Imaris software by quantifying a surface created for tubulin.

### Quantitative PCR

Cells were infected with equal multiplicities of infection (MOIs), and reverse transcription-polymerase chain reaction (RT-PCR) was conducted to assess the relative expression of the late reverse transcription (Late RT) and 2-LTR products. GAPDH was employed as the housekeeping gene for normalization. Initially, the virus was treated with DNase for 1 h at 37°C prior to infection. Genomic DNA from cells was then extracted 20 h post-infection following the protocol of the DNeasy Blood and Tissue Kit (Qiagen). Equal amounts of DNA were utilized for each sample and were run in triplicate during the RT-PCR analysis. The following primer sets were utilized for 2LTR and LateRT analysis: 2LTR Fwd 5′- AAC TAG GGA ACC CAC TGC TTA AG- 3′; Rev 5′-TCC ACA GAT CAA GGA TAT CTT GTC-3′. LateRT (mCherry) Fwd 5′- GTG AGC AAG GGC GAG GAG GAT-3′; Rev 5′- CTT GTA CAG CTC CAT GCC-3′.

### Statistical analysis

GraphPad Prism version 6.00 (GraphPad Software, Inc.) was employed for statistical analysis and to make graphs. Statistical significance was assessed using a one-tailed *t*-test. *P* < 0.05 was considered significant. Data were presented as mean ± SEM.
